# Mutual and Opposing Responses of Carabid Beetles and Predatory Wasps to Local and Landscape Factors in Vineyards

**DOI:** 10.3390/insects11110746

**Published:** 2020-10-30

**Authors:** Deniz Uzman, Martin H. Entling, Ilona Leyer, Annette Reineke

**Affiliations:** 1 Department of Crop Protection, Hochschule Geisenheim University, Von-Lade-Str. 1, 65366 Geisenheim, Germany; Annette.Reineke@hs-gm.de; 2 iES Landau, Institute of Environmental Science, University of Koblenz-Landau, Fortstraße 7, 76829 Landau, Germany; entling@uni-landau.de; 3 Department of Applied Ecology, Hochschule Geisenheim University, Von-Lade-Str. 1, 65366 Geisenheim, Germany; ilona.leyer@hs-gm.de

**Keywords:** organic farming, predators, viticulture, solitary wasps, carabids, vegetation, landscape structure

## Abstract

**Simple Summary:**

The aim of this study was to contribute to closing knowledge gaps on managing vineyards and viticultural landscapes in order to support insect diversity and abundance. We studied two different groups of predating insects, carabid beetles and cavity-nesting wasps, in organically and conventionally managed vineyards in Germany. Effects of surrounding landscapes and vegetation structure within vineyards were evaluated. No differences in species richness and abundance of carabid beetles and cavity-nesting wasps were found between organic and conventional management. Enhanced vegetation cover was positively correlated with carabids and negatively with wasps. High covers of annual crops in the surrounding landscape led to fewer species and individuals of both groups. The results underline the importance of insect-friendly management, especially in intensely farmed landscapes.

**Abstract:**

Preserving agro-biodiversity is one of the main means at the moment to counteract the global biodiversity crisis. Vineyard inter-rows offer vegetation covers which could function as foraging grounds for arthropods. Furthermore, organic management and enhanced landscape complexity often support biodiversity. Here, species richness and abundance of two groups of arthropod predators in vineyards were studied. Fifteen pairs of organically and conventionally managed vineyards were chosen along a gradient of landscape complexity in Rhine-Hesse, Germany. Carabid beetles were sampled using pitfall traps and cavity-nesting wasps with trap nests, respectively. Proportions of different land-use types surrounding the vineyards were calculated and inter-row vegetation cover was characterized. Species richness and abundances of both predator groups were not significantly affected by the management system. Likewise, increased cover of semi-natural habitats in the surrounding landscape did not promote their diversity or abundance. Instead, the increasing cover of annual crops diminished both groups. Cavity-nesting wasps profited from dense inter-row vegetation cover, while carabids were disadvantaged. The results indicate that distinct taxa within the same trophic group can respond oppositely to vineyard management. Thus, inter-row vegetation management with densely and sparsely vegetated elements might be best to support predator diversity. Overall, our results suggest that organic viticulture alone is insufficient to assist the studied insect groups, and that other local and landscape management options are needed for their protection.

## 1. Introduction

Agricultural intensification is a major driver of the global biodiversity crisis and worldwide declines in insects [1,2,3]. Thus, a change in land-use practices is pivotal to counteract these developments [4,5,6]. Within agricultural landscapes dominated by annual crops, semi-natural habitat elements represent relatively stable habitats. They also offer additional nesting, feeding and overwintering sites for invertebrates and natural enemies in particular (e.g., [7,8,9]). In-between highly disturbed annual cropping systems and undisturbed semi-natural habitat elements (SNHs), perennial crops like vineyards or orchards could theoretically constitute relatively stable habitats with intermediate disturbance intensities [10,11,12]. Viticultural landscapes and associated dry grassland habitat elements have been of conservation interest for endangered, xerothermic or rare species for quite some time, such as spiders [13], butterflies [14,15,16] and plants [17]. More recently, their potential to contribute to a generally enhanced agrobiodiversity and sustainability has been a focus of research [12,18,19].

Proposed management actions to enhance biodiversity and ecosystem services within vineyards are similar to the recommendations for annual cropping systems [6]. In viticulture, they encompass mostly organic management, provision of SNHs and their connectivity on a landscape scale and the vegetation and soil management of inter-rows [10,11,18]. The effects of organic management and landscape structure on agrobiodiversity have been intensively studied and reviewed in the past (e.g., [20,21]). Most of the studies were conducted in annual crops or grassland with focuses on some popular taxa, such as carabids, spiders or pollinators. This leaves knowledge gaps, as that information cannot be directly transferred to perennial crops like grapevines. Organic management can potentially alleviate negative impacts of intensive agriculture through direct and indirect effects. Under conventional management, applications of pesticides can directly cause mortalities. Indirectly, feeding resources such as weeds or herbivores are reduced [22] In consequence, organic management is often accompanied with an increase in biodiversity and abundance of arthropods. Effect sizes and directions depend on taxonomic groups, functional traits and landscape context [20,21,23,24]. Further, positive effects of organic management can be much stronger in annual than in perennial systems [21,24]. Predators or natural enemies are expected to benefit from organic management by an increase of abundance, diversity or both [21,24]. Responses of individual species within taxonomic groups and in-between studies can be highly variable [20]. Benefits of organic management can become stronger, vanish or be reversed depending on the taxonomic resolution [22,25,26] or the landscape context [23,24].

At the landscape scale, an enhancement of SNH elements and associated landscape complexity within agricultural landscapes supports several ecosystem services [9]. Furthermore, it promotes numerous pollinators and natural enemies [27]. However, again, effect sizes and directions are trait- and crop dependent; e.g., perennial crops might profit less from enhanced landscape complexity in terms of biodiversity [27]. It is also important to take the connectivity of SNH elements and crop fields into account [8]. The connectivity of habitat elements is important for arthropod populations and dispersal [28]. The proximity of SNH elements to respective crops affects colonization by arthropods [28]. Moreover, SNHs are favored overwintering habitats for predators [29]. An increased habitat connectivity in the landscape can therefore enhance ecosystem services, such as aphid suppression and pollination [30,31]. On the other hand, adverse effects of SNH elements and their connectivity are possible. For instance, they can act as dispersal barriers for carabid beetles [8,32].

On a local scale, the vegetation cover in the vineyard inter-rows can alter their ecological value. Biodiversity in vineyards is expected to increase with extensive vegetation management [12,33]. In general, vegetation cover influences ground-dwelling communities of arthropod predators [34]. In vineyards, the management of inter-row cover crops has been shown to alter soil invertebrate communities [35]. Specifically, changes in vegetation alter the availability of feeding and nesting resources and microclimatic conditions. Moreover, the vegetation cover can offer shelter from predators and weather [36,37,38]. However, not only ground-dwelling predators are affected: plant diversity facilitates overall generalist predators [39], and more specialized natural enemies, such as cavity-nesting wasps [40,41].

Here, we studied the combined and interactive effects of organic management, landscape structure and inter-row vegetation on predatory insects in vineyards. Available data for temperate viticultural systems are scarce. This makes multi-taxon approaches covering organisms with different life-histories even more important, as each group of natural enemies can have distinct habitat requirements [42,43,44]. For the present study, we selected two predatory insect groups with contrasting life histories: the commonly assessed carabid beetles and—the less frequently assessed—cavity-nesting wasps. While carabid beetles are soil-dwelling predators, cavity-nesting wasps are usually flight-active. In addition, they differ in their dispersal abilities, and food and nesting requirements [45,46].

Carabid beetles are key predators in many types of farmland [36,47,48]. For the most part, they are polyphagous predators and can thus potentially act as ground-dwelling biocontrol agents of insect pests or weeds [49,50]. There are spring and autumn breeders. Carabids breeding in spring overwinter, as adults and autumn breeders hibernate primarily as larvae [46]. For overwintering, they depend on stable habitats, such as field margins or hedges [51,52]. Their dispersal ability is determined by their wing-form and their body size. Hind wings can be reduced (brachypterous forms), fully developed (macropterous forms) or dimorphic [46,53]. Hence, species with reduced wings are locally more restricted than macropterous species [54].

Cavity-nesting wasps feed on nectar and pollen as adults, but they also hunt small arthropods as food for their offspring [45]. Thus, cavity-nesting wasps are multi-habitat users: They use woody semi-natural habitats for nesting in cavities, arable crops for hunting and crops or flower-rich habitat elements to feed on floral nectar [55,56]. The access to these resources determines their survival in agricultural landscapes [57]. Similarly to cavity-nesting bees, they build brood cells during summer within above-ground cavities such as dead wood or hollow stems [58]. Each cell is closed after eggs and food provisions are deposited [59]. Within the cells they overwinter as larvae or pupae and hatch in the next vegetation period. Most species are univoltine, but there are also bivoltine or partially bivoltine species [60,61]. Within this nesting guild, there are different hunting strategies [58]. Eumenid wasps (*Discoelius* sp., *Ancistrocerus* sp., *Microdynerus* sp. or *Allodynerus* sp.) often prey on pest caterpillars, such as the larvae of apple tortricids [58,62,63]. Sphecid and pompilid wasps such as *Trypoxylon* sp. and *Dipogon* sp. prey on spiders [58]. There are limited data on their foraging and dispersal ranges, but they probably forage and disperse within radiuses of several hundred meters [63,64].

To compare effects of different management actions on carabid beetles and cavity-nesting wasps, 15 pairs of conventionally and organically managed vineyards in a German grapevine growing area were studied. The vineyard pairs were placed along a gradient of landscape complexity. Vegetation characteristics within the inter-rows were assessed. We hypothesized that (i) increased vegetation cover in the vineyard inter-rows would support both taxonomic groups with the additionally offered resources. We further hypothesized that (ii) predators in vineyards would profit from organic management, and that (iii) enhanced landscape complexity would have stronger effects on cavity-nesting wasps than on beetles due to their dependency on woody nesting sites and their higher dispersal capacity and foraging range.

## 2. Materials and Methods

### 2.1. Study Sites

The study took place in 15 pairs of organically and conventionally managed vineyards in Rhine-Hesse, Germany (see [65,66,67]). Conventional management was applied according to the standards of integrated pest management (EU Directive 2009/128/EC). The majority of the organic wineries were certified by “Ecovin,” which is a German federal association of organic viticulture. The main differences between conventional and organic vineyard management in regard to the agrochemicals used are that synthetic pesticides are prohibited under organic management. Instead, copper-bearing products are commonly used. Further, mineral fertilizers and the application of herbicides for the area beneath vines are illicit in organic vineyards [68]. Vineyard pairs were at least 1000 m apart from each other. Distances between each pair’s organic and conventional vineyard ranged between 16 and 512 m (see Figure 1 in [66]). Within sites, every other inter-row was kept with a permanent vegetation cover while the other one was tilled once or twice per vegetation season (see [67] for more details on vegetation within sites). In 2016, some vineyards were not tilled due to heavy rainfall, but the latest tillage events took place in 2015.

### 2.2. Landscape Analysis

Landscape was characterized within a radius of 1000 m around each vineyard using aerial photographs and QGIS 2.18.9 (https://qgis.org/). Within this radius, the respective percentages of area covered by annual crops (such as winter wheat, canola, sugar beet), perennial crops (such as stone fruits and pome fruits), viticulture and SNH (split in herbaceous and woody habitat elements) was calculated. Further, proximities of the vineyards to the next SNH element were determined as a measure of connectivity. These proximities were again split up in proximities to either woody or herbaceous habitat elements.

### 2.3. Vegetation Parameters

At each site, vegetation in the inter-rows was characterized at three time points during the vegetation period of 2016 (March, June, September). On each occasion, two central, adjacent inter-rows were sampled to account for the alternating tillage treatment. A rectangle of 0.5 × 2 m (1 m²) was used, which was placed twice per inter-row (15 and 25 m from the field edge). In each rectangle, vegetation cover (%) was estimated and vegetation height (cm) was measured. Mean values per vineyard were calculated. Vegetation cover and height represent aspects of the vegetation structure, which is an important variable in structuring arthropod predator communities [69].

### 2.4. Arthropod Sampling

#### 2.4.1. Cavity-Nesting Wasps

Cavity-nesting wasps were sampled using the trap nest approach applied in [67]. In short, artificial trap nests filled with reed were attached to two central vineyard poles from March until September 2016. After recollection, nesting tubes (reed straws containing nests) were isolated and hibernated at 4 °C until February 2017. An outside incubation period followed until specimens had hatched. Hatched adults were identified on the species level. Species richness and abundance per vineyard were calculated. Abundance was defined as the number of nesting tubes (not brood cells). At one conventional vineyard, trap nests were lost to the harvesting machine. This vineyard was omitted from analysis.

#### 2.4.2. Carabid Beetles

Carabids were caught by the same pitfall traps used in [66]. In short, three pitfall traps were placed in each vineyard. Two pitfall traps were placed in two adjacent inter-rows and the other one beneath the vine rows. Distances between traps were 5 m. Plastic cups with a diameter of 6.8 cm were used. Traps were filled at 2/3 volume with 25% propylene glycol and a few drops of odorless detergent to reduce surface tension. Traps were active for 2 subsequent periods of 9 days in May 2016 (3rd/4th–11th/12th and 11th/12th–19th/20th of May). Carabid beetles were identified to the species level. Data were pooled over both periods and species richness and individual numbers per vineyard were calculated. The number of carabid individuals caught by pitfall traps reflects both abundance and activity of species [46]; however, we will refer to these numbers as abundance. From one conventional vineyard, three traps were missing and the vineyard was omitted from analyses. At one organic vineyard, one trap was lost during the second sampling period. For this vineyard, carabid abundance and species richness are based on five instead of six pitfall traps in total.

### 2.5. Data Analysis

Four response variables were obtained: (i) species richness and (ii) abundance of solitary wasps, and (iii) species richness and (iv) abundance of carabid beetles. Stepwise backward model selection of appropriate generalized linear mixed-effect models (GLMMs) was chosen to identify drivers of these two predator groups. Before model selection, the explanatory variable set was reduced. Initially, the set encompassed all landscape parameters (SNH %, herbaceous elements %, woody elements %, annual crops %, perennial crops %, viticulture % and proximities to the next SNH/woody/herbaceous habitat element), vegetation characteristics (vegetation height per m², vegetation cover per m² and flower density per m²) and the management system (organic/conventional). This set was reduced by using a PCA and by calculating the variance inflation factor. Variables with a variance inflation factor (VIF) > 2 were excluded from the full model. This resulted in a final variable set of: SNH %, annual crops %, proximity of the next herbaceous habitat (m), vegetation cover % and management system. Vineyard pair was used as random factor. For each response variable, the appropriate distribution was chosen depending on the variable type and adjustment for under- or overdispersion: Poisson for carabid species richness (count data), negative binomial for carabid abundance (count data, poisson with overdispersion) and Conway–Maxwell for cavity-nesting wasp species richness and abundance (count data, poisson with underdispersion). GLMM model structure was Y ~ intercept + management system + SNH [%] + annual crops [%] + proximity to the next herbaceous habitat (m) + vegetation cover [%] + (1|“pair”). Model selections were done manually based on Chi-Square statistics using the MuMIn: drop1-function via the Akaike information criterion (AICc). The most parsimonious models are presented in the results section. Model fits of the most parsimonious models were checked with diagnostic plots and conditional and marginal R²s were calculated [70] (package: sjstats).

For all analyses, R Version 3.5.3 [71] was used with the packages reshape2 [72], dplyr [73] for data handling, glmmTMB [74] and sjstats [70] for model building, MuMIn [75] for model selection and ggplot2 [76] for plotting. Full data are provided as Supplementary Materials (Table S1).

## 3. Results

In total, 14 wasp species in 128 nesting tubes were collected. The most abundant of the 14 recorded wasp species were *Ancistrocerus parietinus* (family: Vespidae, 39 nests), *Trypoxylon figulus* (family: Crabronidae, 19 nests) and *Ancistrocerus antilope* (family: Vespidae, 13 nests). Three of the recorded wasp species were threatened in Germany, *Discoelius zonalis*, *Discoelius doufourii* and *Microdynerus longicollis* (family of all three: Vespidae, Table A1). In the pitfall traps, 1107 carabid individuals were caught that belong to 40 species (family: Carabidae). The most abundant species were *Amara aenea* and *Nebria brevicollis* (both 159 individuals), followed by *Brachinus explodens* (135 individuals). Nine of the recorded carabid species were of conservation concern in Germany (Table A2). Complete species lists of both groups are given in Appendix A.

**Figure 1:**
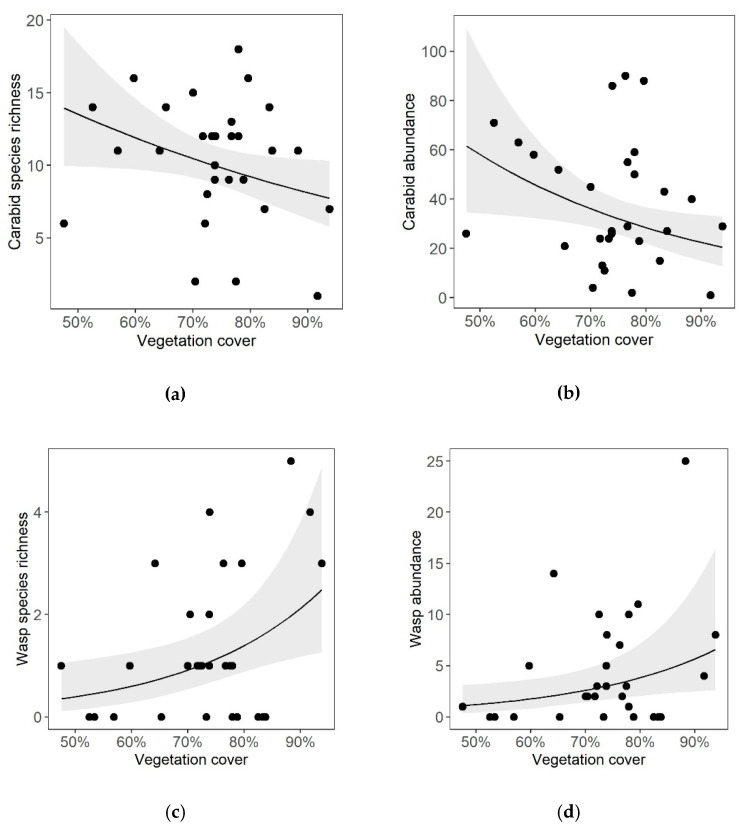
Effects of the inter-row vegetation cover on (**a**) carabid species richness, (**b**) carabid abundance, (**c**) cavity-nesting wasp species richness and (**d**) wasp abundance in 15 organically and conventionally managed vineyards in Rhine-Hesse, Germany (2016). Solid lines represent the prediction curves based on the respective GLMMs (Table 1). Grey ribbons represent confidence intervals of 95%.

Vegetation cover in the inter-rows affected species richness and abundance of both cavity-nesting wasps and carabid beetles, in different directions. Species richness and abundance of cavity-nesting wasps were enhanced by increasing vegetation cover (E = 0.47 ± 0.18, *p* = 0.01; E = 0.43 ± 0.20, *p* = 0.03, respectively, Table 1, Figure 1c,d), while carabid species richness and abundance decreased (E = −0.14 ± 0.07, *p* = 0.04, E = −0.26 ± 0.12, respectively, *p* = 0.02, Table 1, Figure 1a,b).

**Figure 2:**
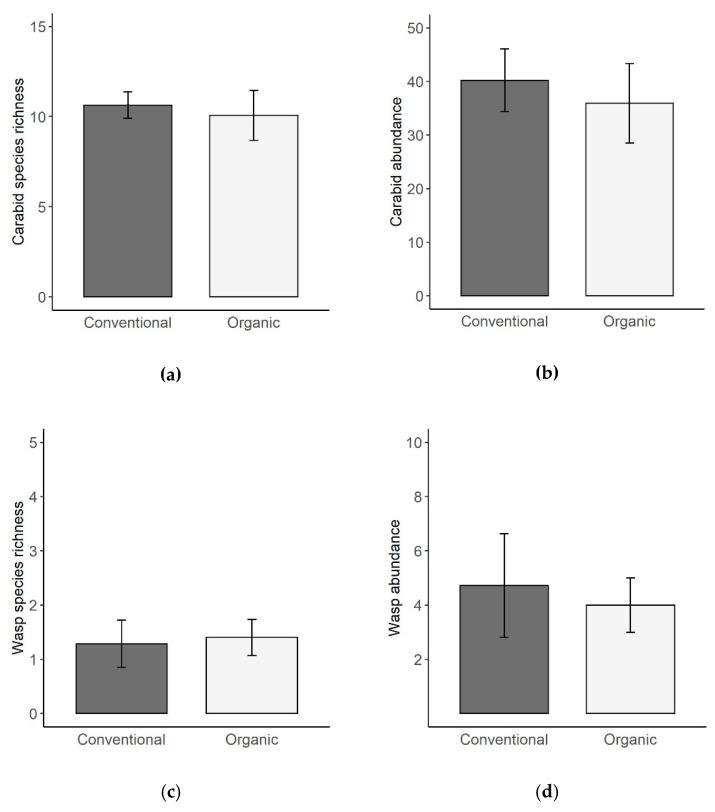
Comparison of species richness and abundance of carabid beetles and cavity-nesting wasps in organic and conventional vineyards collected from March until September 2016 (wasps) and in May 2016 (carabids). Shown are (**a**) species richness of carabid beetles, (**b**) abundance of carabid beetles, (**c**) species richness of cavity-nesting wasps and (**d**) abundance of cavity-nesting wasps. For all parameters, means and standard errors (SE) per vineyard are displayed.

Organic versus conventional management did not have any significant effect on carabid or wasp species richness or abundance (Figure 2a–d). The variable management system was omitted during model selection for each of the four response variables (Table 1).

**Figure 3:**
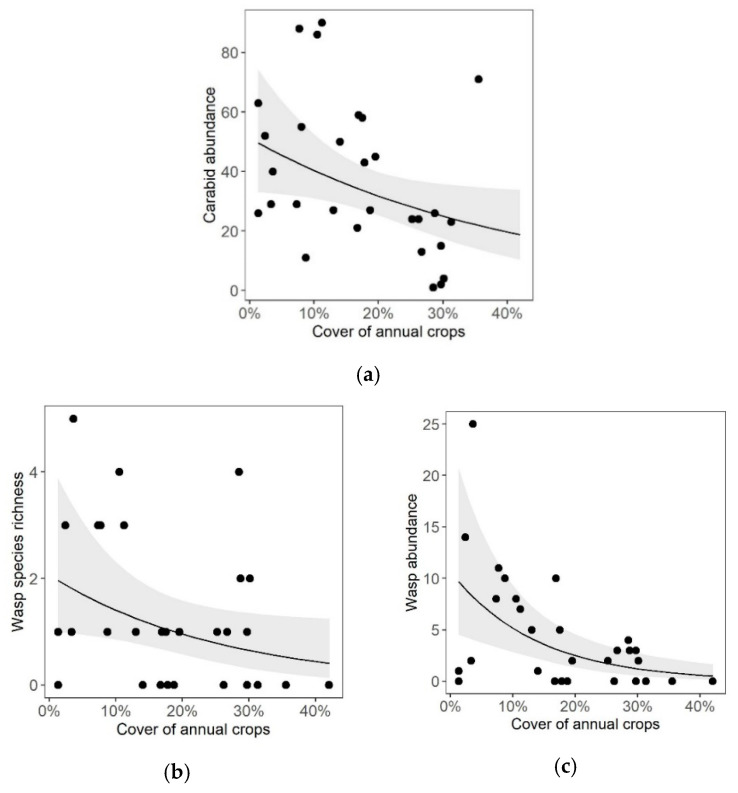
Effects of the proportion of annual crop cover within a 1000 m radius around vineyards on (**a**) carabid abundance and on (**b**) species richness and (**c**) abundance of cavity-nesting wasps in 15 organically and conventionally managed vineyards in Rhine-Hesse, Germany (2016). Solid lines represent the prediction curves based on the respective GLMM (Table 1).

The cover of SNH in the surroundings of the vineyards only had marginal but positive effects on carabid species richness (Table 1). In contrast, the increasing cover of annual crops in the vicinity of vineyards had clear negative effects on both cavity-nesting wasps and carabids: carabid abundance (Table 1, Figure 3a), wasp species richness (Table 1, Figure 3b) and abundance (Table 1, Figure 3c) were reduced in vineyards with high percentages of cover of annual crops in the surroundings. Carabid species richness was not affected by the area covered by annual crops (Table 1).

**Figure 4:**
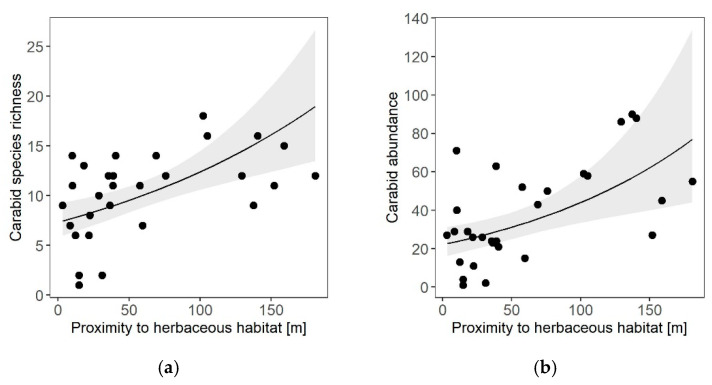
Effects of the proximity of the next herbaceous semi-natural habitat element on (**a**) carabid species richness and (**b**) carabid abundance in 15 organically and conventionally managed vineyards in Rhine-Hesse, Germany (2016). Solid lines represent the prediction curves based on the respective GLMM (Table 1). Grey ribbons represent confidence intervals of 95%.

Species richness and abundance of carabids increased with increasing isolation from herbaceous habitats (Table 1, Figure 4a,b), meaning that the further away the next herbaceous habitat was, the more species and individuals caught per vineyard. For cavity-nesting wasps, the proximities of neither herbaceous nor woody habitat elements were relevant during model selection.

## 4. Discussion

### 4.1. Effects of Vineyard Inter-Row Vegetation Cover on Carabid Beetles and Cavity-Nesting Wasps

In this study, carabids and cavity-nesting wasps showed opposite reactions towards the inter-row vegetation cover. While cavity-nesting wasps profited from higher vegetation cover, carabid species richness and abundance decreased. The relationship between carabid communities and vegetation cover is complex. The effect sizes and directions of vegetation structure on carabid beetles can differ between seasons or trophic groups [77]. We expected vegetation cover to enhance feeding and hunting resources for carabid beetles and to provide more favorable microclimatic conditions [37]. However, also the opposite effect can occur [78], which has been observed in vineyards too [79]. Many carabid beetles prefer open vegetation with high light levels. Our third most abundant species *Brachinus explodens* is a xerothermic species that is restricted to the warmest parts of Germany [47]. Thus, higher irradiation at ground level could be the main factor why sparse vegetation cover favors high carabid densities. On the other hand, a negative effect of vegetation cover on carabids can as well be caused by a methodological bias, as a more complex and dense vegetation impedes carabid movement and reduces the probability of falling in the pitfall traps [80,81,82]. In some cases, plant functional composition is more suitable to explain carabid assemblages [38,77]. In our case, an enhancement of vegetation cover in vineyard inter-rows might not necessarily lead to an enhancement of carabid diversity or abundance.

In contrast to the carabids, cavity-nesting wasps profited from enhanced vegetation cover. Neither their species richness nor their abundance could be related to floral diversity and flower richness in the inter-rows [83]. In conclusion, we assume that the vineyards were rather used as hunting sites in order to feed their offspring than as foraging sites for adults. Natural enemies encounter their prey more frequently in complex vegetation and by capturing them more efficiently [84]. Whether or not cavity-nesting wasps are limited by floral resources might depend on the landscape context [57]. If enough floral resources are available outside crops within short flight ranges of less than 200 m [56], enhanced in-field floral availability might not lead to additional positive effects. For example, in boreal habitats, flower richness in clear-cut areas enhanced wasp species richness [85]. Maybe in this environment, neither nesting nor hunting resources were the limiting factor, but floral resources. In our study, floral resources did not promote (cavity-nesting) wasps. This has been observed before [86,87]. Besides, species-specific plant preferences have to be considered when evaluating effectiveness of in-field floral supply [45]. The right choice of inter-row plant species can also promote ecosystem services like parasitism rates [88,89].

### 4.2. Carabid Beetles and Cavity-Nesting Wasps in Organic and Conventional Vineyards

Neither species richness nor abundance of the studied insects were affected by organic or conventional vineyard management. In a meta-analysis, pollinators and predators were supported by organic management, while herbivores and detrivores showed less reactions [21]. However, not only can functional groups differ in their responses towards organic management; the crop type can be pivotal. Strongest positive effects have been observed in cereal crops [24]. Correspondingly, arthropod abundances were increased by 70% in organically managed annual crops, but only by 1% in organic perennial crops when compared to conventional management [21]. This is in line with our results, similarly to Bruggisser et al. [10], who compared different trophic groups in organic and conventional vineyards and found no effects of management system on plant, spider and grasshopper abundance and diversity. In the vineyards studied here, spiders responded positively to organic management, but much less strongly than in annual crops [66]. Accordingly, for perennial crops like grapevines, knowledge obtained from annual cropping systems cannot be transferred one to one. In the presented study, organic vineyards received more frequent plant protection treatments during vegetation season and copper-based fungicide agents were frequently used [65]. Further, even though floral resources were enhanced in organically managed inter-rows, vegetation cover did not differ between management systems [67]. Herbicide application in conventional German viticulture is restricted to the area directly beneath the vines [90]. In organic vineyards, this area is also kept free from vegetation, but mechanically. This is a systematic difference in the use of herbicides compared to arable fields. Overall, a reduced intensity of organic versus conventional management is not as clear in vineyards as in annual crops. Interactions between the management system and landscape context can be expected [91], but in the case of the two predator groups presented, the authors could not find evidence for a clear interactive effect between organic or conventional management and the cover of SNH on a landscape level [83]. Including an interaction in the initial full models (see Section 2.5) either led to the same most parsimonious model, or models without the interaction performed better based on the AICc criterion (delta AICc > 3). Thus, initial models were kept as simple as possible and without interactions.

For carabids in particular, variable responses to organic management in agricultural systems have been reported. The results ranged from slightly enhanced abundance in organic wheat fields [92], to higher species numbers and abundance in organic winter wheat [93] and other cereal crops [94] to no visible effects in cereals [95,96,97]. In a study by Diekötter et al. [97], interactions between organic management and the amount of organically managed fields in the surroundings determined carabid species richness. In vineyards, conventional sites showed higher species richness of brachypterous carabids than organic ones [98]. Higher abundance in conventional soybeans compared to organic counterparts have also been reported [99]. This variety of response patterns of carabid beetles towards the management system leads to the conclusion that other factors might be involved determining abundance and species richness of this arthropod group.

However, even if abundance and species richness of carabids do not seem to be affected by the management system, functional diversity may differ between community assemblages in both management systems [28,98]. Certain carabid species, such as *Amara aenea*, *Harpalus distinguendus* and *Nebria brevicollis*, are associated with organic management in different (annual) cropping systems [78,95,100,101]. This study’s data did not reveal such findings. Abundance of *N. brevicollis* was similar between both management systems (78 individuals in conventional vs. 81 in organic, Table A2). Further, *A. aenea* and *H. distinguendus* were each more than twice as frequent in conventional than in organic vineyards (Table A2). The species found within this study represent species that are associated with viticulture in the Southwest of Germany (e.g., *Ophonus azureus, B. explodens, N. brevicollis, H. tardus* and *H. distinguendus*), but also species that are commonly found in arable fields (*Bembidion lampros, Carabus auratus*), or both (*Amara aenea* and *H. affinis*) [47].

Little is known about effects of general in-field management measures on farmland wasps [86]. To our knowledge, only one study compared cavity-nesting wasps in organic and conventional fields to date [102]. In their case, organic management in wheat supported cavity-nesting wasp abundance and species richness [102]. Contrarily, in the presented study, species richness and abundance did not differ between both systems. Supposedly, this might be related to prey availability. This can be explained using the example of spiders as prey taxa for crabronid and sphecid wasp species. In vineyards, spider abundance does not profit from organic management [10,66,103]. This implies a comparable prey offer between both systems in vineyards. In contrast, more web-building spiders can be found in organic than in conventional cereal fields [104,105]. In the latter study, the spider *Phylloneta impressa* was clearly more abundant in organic wheat. This species, in turn, belongs to the favored prey species of the sphecid wasp *Trypoxylon figulus* [106]. Information on the distribution of *P. impressa* in conventional versus organic vineyards is to our knowledge not available.

### 4.3. Landscape Effects on Carabid Beetles and Cavity-Nesting Wasps

Contrary to the initial hypothesis, enhanced landscape complexity (in terms of SNH cover) did not promote abundance or species richness of either carabids or cavity-nesting wasps. Instead, carabid abundance and wasp species richness and abundance within the vineyards were reduced with increasing cover of annual crop fields. Previous studies have reported a wide range of response patterns by carabid beetles towards different landscape contexts. Enhanced landscape complexity promoted carabid beetles [107], did not affect them [108] or even reduced them [109]. In turn, high cover of annual crops enhanced carabid abundance in cereals in France [110] and across Europe [111]. Dynamics and composition of carabid populations can vary considerably between seasons and thereby the momentary importance of landscape structure [112]. In general, most of the carabid species that are common in vineyards are adapted to cultivated soil and even depend on it to complete their life cycle [113]. In the presented study, higher carabid abundance and species richness were observed in vineyards that were more isolated from herbaceous SNH elements. In a similar study in France, Rusch et al. detected a negative relationship between enhanced landscape complexity and carabid numbers in vineyards. They suggested that SNH elements possibly act as sinks for carabids in viticultural landscapes, thereby reducing their abundance [109]. Alternatively, antagonistic interactions between carabids and their enemies such as birds in complex landscapes could explain the observed pattern [114].

Due to their different hunting strategies, trophic guilds within cavity-nesting wasps can determine their responses towards different landscape contexts. Depending on the functional group within cavity-nesting wasps, different landscape characteristics and their suitability as hunting and foraging grounds determine their abundances [115]. For instance, arable fields can be considered as decent hunting and foraging habitats for spider-predating wasps [64], while caterpillar hunting wasps can find more prey in the proximity of wildflower strips and sites with a high amount of grassland cover [115]. In the presented study, high cover of arable crops negatively affected cavity-nesting wasps. Presumably, arable crops do not represent ideal hunting grounds for the species we found. Even though spider-hunting *T. figulus* was present, the majority of cavity-nesting wasps belonged to the caterpillar-hunting guild.

Surprisingly, we found no evidence of nesting limitations for cavity-nesting wasps in vineyards. The proximity of woody habitats with natural cavities is strongly enhancing cavity-nesting wasps [56,116]. Moreover, cavity-nesting bees strongly depended on the proximity of woody habitats within the vineyards studied here [67]. Supposedly, low abundance of cavity-nesting wasps and the missing link to habitat connectivity are caused by methodological reasons or the low number of replicates. Trap nest are very dynamic systems with inter-trophic interactions, where bees and wasps compete with each other [59]. Possibly, cavity-nesting bees were strong competitors in this study (see Section 4.4).

In summary, we found no evidence for beneficial effects of increased landscape complexity or connectivity on natural enemies in vineyards. This is in line with the results from a recent study in France [117]. Instead, the cover of annual crops reduced two guilds of natural enemies within a perennial cropping system. This emphasizes that agricultural intensification in regard to annual crops on a landscape-scale can cause arthropod declines in nearby land-use types: in protected areas [118], grassland and forest patches [3] and, in our case, viticulture.

### 4.4. Relative Biocontrol Potential of Cavity-Nesting Wasps in Viticulture

A dense vegetation cover in vineyard inter-rows could easily support cavity-nesting wasps. Moreover, it still needs to be ensured that all habitat types needed for their lifecycle are available within small ranges. Data about their foraging range is scarce, but there is evidence that it might be limited to a few hundred meters [63]. Around 2/3 of the individuals we caught belonged to the Eumenid species which often prey on (caterpillar) pest larvae [58]. They might be useful as biocontrol agents in vineyards. For example, *Ancistrocerus gazella* has been reported to prey on tortricid larvae [62]. Additionally, pests such as the European grapevine moth *Lobesia botrana* and *Eupoecilia ambiguella* belong to this group [119]. Their efficacy against leafrollers has been tested on in fruit crops [120]. Further, *A. parietinus*, our most abundant wasp species, preys on Chrysomelid larvae [58], some of which can act as pest species in table grapes [121,122]. The assessment of their concrete biocontrol potential in vine needs to be further investigated.

It has to be pointed out that for both assessed groups, relatively low individual numbers were caught when compared to other studies. Low carabid caches in vineyards had also been mentioned by [109]. The abundance of cavity-nesting wasps with 128 nests was considerably lower than for cavity-nesting bees with more than 900 nests within the same trap nests [67]. In other trap-nest studies, adverse relationships between bee and wasp nest numbers were reported (e.g., [116,123]). These shifts in the bee–wasp ratio can be caused by trophic interactions between them and their parasitoids [59]. Possibly, vineyards are poor habitats for cavity-nesting wasps, being more sensitive towards plant protection products or an overlap of their main activity season and an increased intensity of grape cultivation starting in July. There are more data needed to investigate wasps in this specific habitat.

## 5. Conclusions

We presented a study in which two different guilds of predatory insects were investigated within the same vineyard sites. Carabid beetles and cavity-nesting wasps exhibited mutual and opposing reactions to local management and landscape context in this perennial crop. Diversity and abundance of both groups showed no clear differences between organic and conventional vineyard management. Further, they did not profit from enhanced landscape complexity and connectivity. In turn, high cover of arable cropping systems on a landscape scale reduced their species richness and abundance. Carabids and cavity-nesting wasps had opposing preferences in regard to inter-row vegetation cover. In summary, for the two taxonomic groups assessed here, organic management alone might have limited potential to enhance predator diversity in vineyards. As the insects were negatively affected by crop area rather than being positively affected by SNH, our results suggest that negative effects of arable land cannot simply be overcome by the introduction of small areas of SNH. Supposedly, agri-environmental schemes for insect biodiversity in viticulture could thus become more efficient when adapted to the landscape context of the specific area.

## Figures and Tables

**Table 1:** Effects of landscape structure, local vegetation and management system on species richness and abundance of carabid beetles and cavity-nesting wasps. The presented estimates and p-values were obtained from the most parsimonious model after stepwise model selection (full model: Y ~intercept + SNH % + annual crops % + proximity to the next herbaceous habitat (m) + vegetation cover % + management system, with vineyard pair as random factor). Management system was omitted during model selection for each response variable. Marginal R² gives explained variation without and conditional R² with the random factor. The chosen statistical distribution for the respective GLMM is given. Significant predictors (*p* < 0.05) are printed in bold.

Response Variable	Predictors	Estimate ± SE	*p*-Value	R²_cond/marg_	Distribu-tion	AICc	dAICc (to Full Model)	AICc (Full Model)
Carabid beetles								
Species richness	(Intercept)	2.31 ± 0.07	<2 × 10^−16^ (***)	0.38/0.44	Poisson	167.8	3.9	171.7
	SNH %	0.15 ± 0.08	0.05					
	**Vegetation cover %**	−0.14 ± 0.07	0.04 (*)					
	**Distance to herbaceous habitat**	0.28 ± 0.08	<0.001 (***)					
Abundance	(Intercept)	3.51 ± 0.11	<2 × 10^−16^ (***)	0.50/0.50	Negative binomial	262.1	3.6	265.7
	**Annual crops %**	−0.27 ± 0.13	0.03 (*)					
	**Vegetation cover %**	−0.26 ± 0.12	0.02 (*)					
	**Distance to herbaceous habitat**	0.36 ± 0.12	0.002 (**)					
Cavity-nesting wasps								
Species richness	(Intercept)	0.04 ± 0.25	0.87	0.37/0.50	Conway–Maxwell	90.4	3.7	94.1
	**Annual crops %**	−0.44 ± 0.21	0.04 (*)					
	**Vegetation cover %**	0.47 ± 0.18	0.01 (*)					
Abundance	(Intercept)	1.07 ± 0.30	<0.001 (***)	0.02/0.02	Conway–Maxwell	141.6	5.6	147.2
	**Annual %**	−0.82 ± 0.22	<0.001 (***)					
	**Vegetation cover %**	0.43 ± 0.20	0.03 (*)					

## Supplementary Materials

The following are available online at https://www.mdpi.com/2075-4450/11/11/746/s1. Table S1: Raw data for the present study.

## Appendix A

**Table A1:** Abundances of all cavity-nesting wasp species collected in 15 organically and 15 conventionally managed vineyard pairs in Rhine-Hesse, Germany (2016).

Family	Species	Management System		Total
		Conventional	Organic	
*Crabronidae*	*Trypoxylon attentuatum-deceptorium*	3	0	3
	*Trypoxylon clavicerum*	3	1	4
	*Trypoxylon figulus*	11	8	19
	*Trypoxylon spec.*	6	7	13
*Pompilidae*	*Dipogon spec.*	4	1	5
	*Dipogon subintermedium*	2	1	3
*Vespidae*	*Allodynerus rossii*	1	0	1
	*Ancistrocerus antilope*	0	13	13
	*Ancistrocerus claripennis*	1	4	5
	*Ancistrocerus gazella*	4	2	6
	*Ancistrocerus nigricornis*	6	0	6
	*Ancistrocerus parietinus*	22	17	45
	*Discoelius doufourii*	0	1	1
	*Discoelius zonalis*	1	1	2
	*Microdynerus longicollis*	0	3	3
	*Microdynerus timidus*	2	3	5
	Sum	66	62	128

**Table A2:** Abundances of all carabid species collected in 15 organically and 15 conventionally managed vineyard pairs in Rhine-Hesse, Germany (2016).

Family	Species	Management System		Total
		Conventional	Organic	
*Carabidae*	*Acupalpus interstitialis*	2	2	4
	*Acupalpus meridianus*	3	8	11
	*Amara aenea*	109	50	159
	*Amara anthobia*		12	12
	*Amara cf convexior*		1	1
	*Amara familiaris*	3	5	8
	*Amara lunicollis*	1		1
	*Amara ovata*	2	11	13
	*Amara similata*	1	2	3
	*Anchomenus dorsalis*	1	1	2
	*Anisodactylus binotatus*	1		1
	*Asaphidion flavipes*	1		1
	*Badister bullatus*	1		1
	*Badister sodalis*		2	2
	*Bembidion lampros*	7	10	17
	*Bembidion properans*		3	3
	*Bembidion quadrimaculatum*	1		1
	*Brachinus crepitans*	10	31	41
	*Brachinus explodens*	60	75	135
	*Callistus lunatus*		2	2
	*Carabus auratus*	6	8	14
	*Carabus monilis*	1		1
	*Cicindela campestris*	10	1	11
	*Harpalus affinis*	46	31	77
	*Harpalus atratus*	3		3
	*Harpalus dimidiatus*	18	23	41
	*Harpalus distinguendus*	83	24	107
	*Harpalus pumilus*	6	13	19
	*Harpalus subcylindricus*	1		1
	*Harpalus tardus*	26	40	66
	*Leistus spinibaris*	3	1	4
	*Microlestes maurus*	13	16	29
	*Microlestes minutulus*	32	47	79
	*Nebria brevicollis*	78	81	159
	*Nebria salina*	4	7	11
	*Notiophilus aestuans*	4	5	9
	*Ophonus azureus*	24	16	40
	*Platynus dorsalis*	2	7	9
	*Poecilus cupreus*	3	4	7
	*Semiophonus signaticornis*	1		1
